# Optical Data Compression in Time Stretch Imaging

**DOI:** 10.1371/journal.pone.0125106

**Published:** 2015-04-23

**Authors:** Claire Lifan Chen, Ata Mahjoubfar, Bahram Jalali

**Affiliations:** 1 Department of Electrical Engineering, University of California Los Angeles, Los Angeles, California, United States of America; 2 California NanoSystems Institute, Los Angeles, California, United States of America; 3 Department of Bioengineering, University of California Los Angeles, Los Angeles, California, United States of America; University of California, San Francisco, UNITED STATES

## Abstract

Time stretch imaging offers real-time image acquisition at millions of frames per second and subnanosecond shutter speed, and has enabled detection of rare cancer cells in blood with record throughput and specificity. An unintended consequence of high throughput image acquisition is the massive amount of digital data generated by the instrument. Here we report the first experimental demonstration of real-time optical image compression applied to time stretch imaging. By exploiting the sparsity of the image, we reduce the number of samples and the amount of data generated by the time stretch camera in our proof-of-concept experiments by about three times. Optical data compression addresses the big data predicament in such systems.

## Introduction

Big data is a broad and popular topic today. The traditional definition refers to the massive amount of data generated in banking, social media, healthcare, and by networked sensors known as the “internet of things”. However, big data is also a challenge in biomedical and scientific instruments [1]. High-throughput real-time instruments are needed to acquire large data sets and to detect and classify rare events. Examples include the time stretch camera [2–12]—a MHz-frame-rate bright-field imager, and the fluorescence imaging using radio frequency-tagged excitation (FIRE)—an ultra-high-frame-rate fluorescent camera for biological imaging [13]. The record throughputs of these instruments have enabled the discovery of optical rogue waves [14], the detection of cancer cells in blood with false positive rate of one cell in a million [15], and the highest performance analog-to-digital converter ever reported [16].

These instruments produce a torrent of data that overwhelms their data acquisition and processing backend. For example, the time stretch imager captures images at roughly one hundred million scans per second with each scan containing about one thousand samples [17, 18]. Assuming each of these samples is digitized with a typical 8 bits of accuracy, time stretch microscopy (STEAM) produces 0.8 Tbit of data per second. Detecting rare events such as cancer cells or rogue signals requires that data be recorded continuously and for a long time to catch the rare events. The need to compress massive volumes of data in real-time has fueled interest in nonuniform time stretch transformation that takes advantage of sparsity in physical signals to achieve both bandwidth compression as well as reduction in the temporal length [1, 19–22]. The aim of this technique is to transform a signal such that its intensity matches not only the digitizer’s bandwidth, but also its temporal record length. The latter is typically limited by the digitizer’s storage capacity.

## Methods

**Fig 1:**
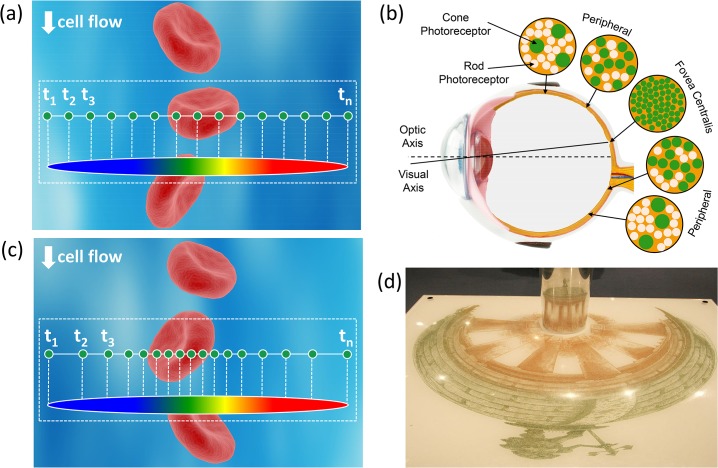
Illustration of warped stretch transform in imaging. (a) The field of view consists of a cell against the background such as a flow channel or a microscope slide. Illumination by an optical pulse that is diffracted into a one-dimensional rainbow maps one dimension of the space into the optical spectrum. The other dimension is scanned by the cell flow through the rainbow. In the conventional time stretch imaging (STEAM), the spectrum is linearly mapped into time using a dispersive optical fiber with a linear group delay. The temporal waveform is then sampled by a digitizer with fixed sampling rate resulting in uniform spatial sampling. But uniform spatial sampling generates superfluous data by oversampling the sparse peripheral sections of the field of view. (b) The human vision is a form of warped imaging system where high sampling resolution is needed in the central vision while coarse resolution can be tolerated in the peripheral vision. (c) Similar functionality can be achieved in STEAM by using a nonlinear group delay profile in the spectrum-to-time mapping process resulting in a nonuniform sampling of the line image, assigning more pixels to the information-rich central part of the field of view and less to the low-entropy peripherals. (d) The reconstruction is similar to anamorphic art where the drawn shape is a stretched and warped version of the true object yet the viewer sees the true object upon reflection from a curved mirror. In our system, this unwarping operation is a nonlinear mapping using the inverse space-to-frequency-to-time mapping transfer function.

The basic principle of time stretch imaging (STEAM) involves two steps both performed optically. In the first step, the spectrum of a broadband optical pulse is converted by a spatial disperser into a rainbow that illuminates the target. Therefore, the spatial information (image) of the object is encoded into the spectrum of the resultant reflected or transmitted rainbow pulse. A one-dimensional rainbow is used to acquire a line-scan and two-dimensional image is obtained by scanning the rainbow in the second dimension. For imaging of particles in flow, the motion causes scanning in the second dimension while the rainbow position is fixed (Fig 1A).

In the second step, the spectrum of the image-encoded pulse is mapped into a serial temporal signal that is stretched in time to slow it down such that it can be digitized in real-time [23]. This optically-amplified time-stretched serial stream is detected by a single-pixel photodetector, and the image is reconstructed in the digital domain. Subsequent pulses capture repetitive frames. The laser pulse repetition rate corresponds to the frame rate, and the temporal width of the pulses corresponds to camera’s shutter speed (exposure time). The key innovations in STEAM that enable high-speed real-time imaging are photonic time stretch for digitizing fast images in real-time and optical image amplification for compensating the low number of photons collected during the ultra-short shutter time [24].

Using warped group delay dispersion, it has been shown that one can reshape the spectro-temporal profile of optical signals such that signal envelope’s time-bandwidth product is compressed [1, 19–22]. The compression is achieved through time-stretch dispersive Fourier transform in which the frequency-to-time mapping is intentionally warped, using an engineered group delay dispersion profile, to match the sparsity of the image. This operation causes a frequency-dependent reshaping of the input waveform. Reconstruction (decoding) method depends on whether the information is in the spectral domain amplitude, or in the complex spectrum. In the time stretch camera, the image is encoded into the amplitude of the spectrum of a broadband optical pulse, and reconstruction consists of a time-to-frequency mapping using the inverse of the measured or simulated group delay profile followed by a frequency-to-space mapping. The compression ratio depends on the group delay characteristics and the sparsity of the image [21, 22]. This method offers similar functionality as compressive sampling [25–31] albeit it achieves it via an entirely different approach, namely by reshaping the analog image using warped time-stretch dispersive Fourier transform.

To illustrate the concept in the context of time stretch imaging, we can consider a microscopic field of view consisting of a cell against a background such as a flow channel or a microscope slide (Fig 1). In the time stretch imaging, the object is illuminated by an optical pulse that is diffracted into a one-dimensional rainbow. This encodes one dimension of space into the optical spectrum. The spectrum is then linearly mapped into time using a dispersive optical fiber with a linear group delay. The mapping process from space-to-frequency-to-time is shown in Fig 1A. The linearly stretched temporal waveform is then sampled by a digitizer resulting in uniform spatial sampling. This uniform sampling (also depicted in Fig 1A) generates redundant data by oversampling the sparse peripheral sections of the field of view.

Such a situation evokes comparison to the mammalian eye where central vision requires high resolution while coarse resolution can be tolerated in the peripheral vision (Fig 1B). In the eye, this problem is solved through nonuniform photoreceptor density in the retina. The Fovea section of the retina has a much higher density of photoreceptors than the rest of the retina and is responsible for the high resolution of central vision.

We solve this problem by nonuniform mapping of spectrum into time via a warped group delay. An example of the warped space-to-frequency-to-time mapping is illustrated in the dotted box in Fig 1C. After uniform sampling in time (by a conventional digitizer), this leads to higher sampling density in the central field of view and lower density in the sparse peripheral regions. This is often desirable in cell screening and imaging in microfluidic channels with focusing mechanisms. In these channels, the cells arrive along a few predetermined lanes. By far the most common case is a single lane aligned to the center of the channel, which is typically achieved via hydrodynamic focusing [32]. However, cells or particles may occasionally appear in peripheral regions of the flow channel. Since the probability of this occurring is low, it would be wasteful to assign high sample density to these peripheral regions. One does need to image these regions albeit with coarse resolution for monitoring rare or abnormal events. In the meantime, the higher sample density in central part of the field of view improves the accuracy of determining cellular morphology, and that of biophysical cell measurements such as cellular protein concentration, which have been previously demonstrated with the time stretch imaging modality [3, 6].

The reconstruction is a simple unwarping using the inverse of the group delay. This operation is analogous to the anamorphic art, where the drawn shape is a stretched and warped version of the true object, yet, the viewer sees the true object upon reflection of the painting from a curved mirror (Fig 1D [33]). In the case where the sparsity characteristic of the target is not known, or changes dynamically, a shift of central field of view is needed. Similar to the movement of the eyeball in the mammalian eye, an active mechanism such as a beam steering mirror can be used to relocate the central field of view, and perform the dense sampling in the region of interest.

**Fig 2:**
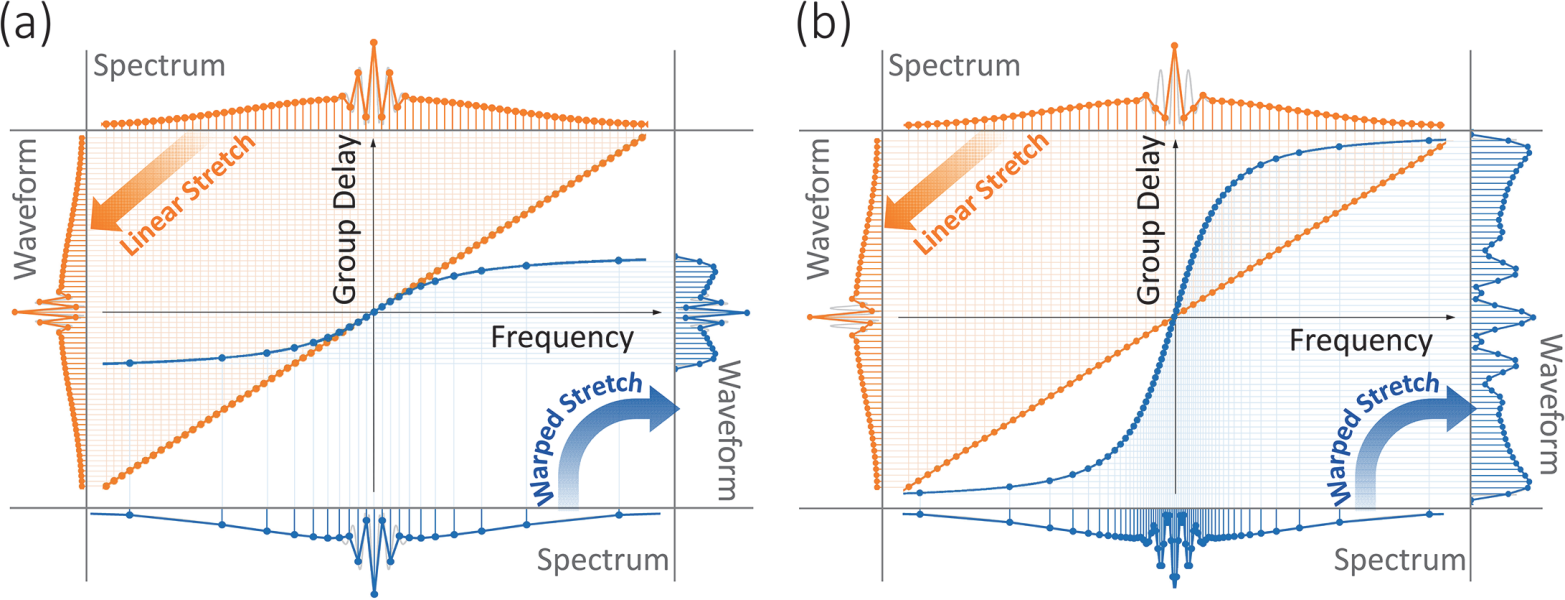
Linear and warped (anamorphic) stretch transforms. The linear group delay profile results in uniform frequency-to-time mapping (orange plots), whereas the warped group delay profile results in nonuniform mapping (blue plots). In both the linear and the warped stretch cases, the stretched waveform is uniformly sampled in time and digitized by an analog-to-digital converter. The amount of linear dispersion is chosen such that the fast features of the waveform are sufficiently slowed down in time to achieve Nyquist sampling. (a) A nonlinear group delay with the same dispersion (slope) at the center of the spectrum as linear case, but shorter total group delay, leads to high sampling resolution in the center of the spectrum and lower resolution at the wings. This keeps the image quality at the central part of the field of view intact, while reducing the quality at the sparse peripheral regions where uniform stretch would have produced redundant samples. Here the digital file size, determined by the overall number of samples, is significantly reduced (compare the waveforms). In other words, data is compressed in optical domain by exploiting its sparsity. (b) A nonlinear group delay profile with higher dispersion (slope) at the center of the spectrum than the linear case, but same total group delay over the bandwidth, leads to a higher spectral resolution in the center of the spectrum and lower resolution at the wings (compare the spectrums). Once again, this improves the image quality at the central region of the field of view by sacrificing the quality at the sparse peripheral regions. Here the file size determined by the overall number of samples in the waveform and the acquisition time remains unchanged. The gray curves show the analog waveforms before sampling for the purpose of comparison.

Different nonlinear group delay profiles result in various types of warped frequency to time mappings. Fig 2A shows a nonuniform group delay profile, which has the same dispersion (slope) as the linear profile in the center of the spectrum, but reduced dispersion at wings. This profile results in data compression by reduction of the overall time duration of the stretched pulses and the number of samples at the expense of lowered spectral resolution in peripheral regions of the spectrum. Fig 2B shows another nonuniform group delay profile, which has the same overall time duration and number of samples as the linear case. This profile redistributes the spectral samples to achieve higher spectral resolution in information-rich central region of the spectrum and lower resolution in sparse peripherals.

**Fig 3:**
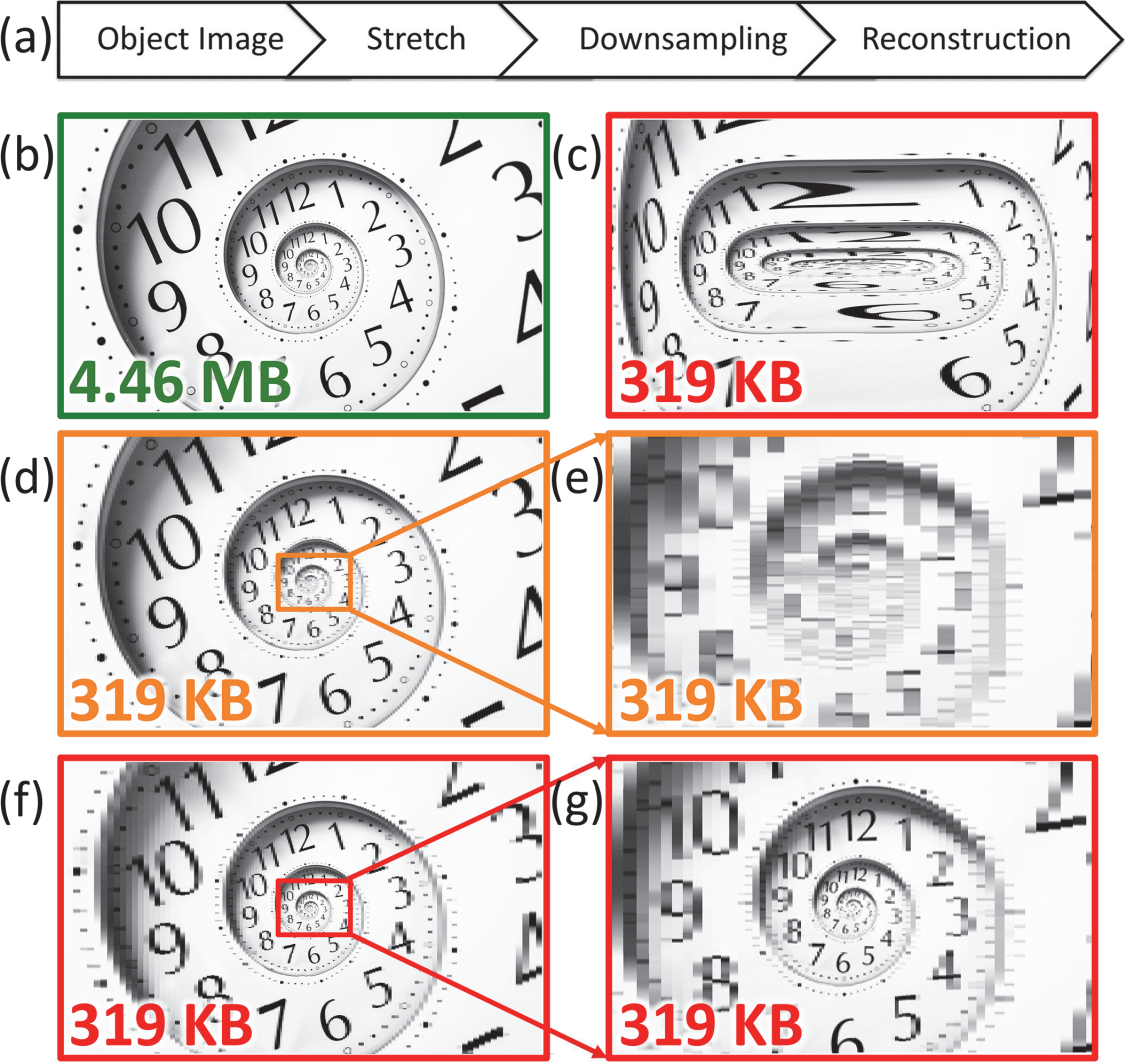
Simulations illustrate the effect of warped stretch transform on a two-dimensional image. The analog reshaping of the image performed in the optical domain by the warped stretch transform is emulated here in the digital domain. (a) The transformation consists of nonuniform stretch in the horizontal direction with the warp stretch profile shown in Fig 2. (b) A sample image with 2800×1672 pixels and 4.46 MB file size is used as the input. It corresponds to an image formed by a linear stretch followed by Nyquist rate sampling. (c) The image is stretched nonuniformly and down-sampled with 200 pixels along the horizontal direction reducing the file size to 319 KB, a compression ratio of 14. The warped stretch profile used here is as shown in Fig 2B causing the sides to be squeezed relative to the center. Hence more samples are assigned to the center than the wings. (d) A uniform stretch with down-sampling can achieve the same file size but the image quality is dramatically lower. (e) While down-sampling is not an issue for the sparse peripherals, it is problematic for the information-rich central part. (f) The reconstruction of the nonuniformly stretched image shown in Fig 3c clearly demonstrates that the resolution is higher at the center where information is rich and lower at the sides where information is much less and relatively not important. (g) The information-rich region at the center is well preserved while maintaining the same sampling rates compared to uniform case without down-sampling shown in Fig 3b.

To help visualize the analog image reshaping performed by warped dispersive stretch and to show how it leads to data compression in imaging, we emulate its effect on a two-dimensional image. As shown in Fig 3A, the image is first stretched and then uniformly down-sampled to achieve data compression, followed by reconstruction (unstretch). By using a nonlinear stretch, the reconstructed image is equivalent to a nonuniformly down-sampled image. Fig 3B shows the original image as if it was generated by a linear dispersion and uniform stretch, and Fig 3C is after nonuniform stretching of the original image in the horizontal direction. The chosen image has higher density of features in the central portion than in the periphery. The warp profile is as indicated in Fig 2B where the peripheral regions are stretched less than the center. Fig 3D is the linearly stretched image after 14:1 down-sampling and reconstruction. As it can be seen in the zoomed-in image 3e, down-sampling has resulted in a loss of resolution. On the other hand, Fig 3F is the nonuniformly stretched image after 14:1 down-sampling followed by reconstruction. Although the final image size is the same, the nonuniformly stretched image has much higher quality in the non-sparse center of field of view (Fig 3G).

Big data problems also appear in light scattering based flow cytometry. There the instrument measures the angular dependence of laser light scattered by particles in flow. The angular scattering profile of microscopic particles significantly depends on their morphological parameters, such as size and shape, and this dependency is widely used in flow cytometry for particle classification [34]. Recently a new spectrally encoded angular light scattering method capable of measuring the continuous angular spectrum has been reported [35]. The warped time-stretch optical data compression technique demonstrated here can also be used for real-time data compression in such optical systems.

## Results

**Fig 4:**
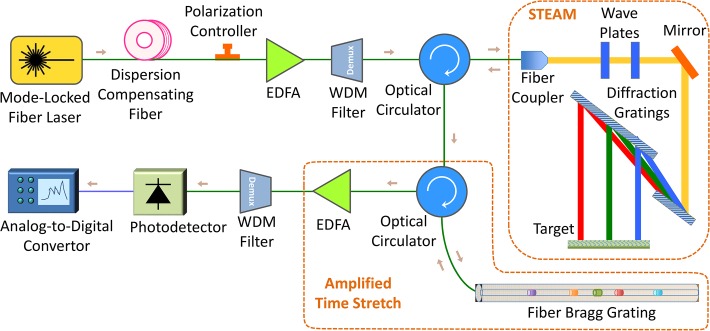
Experimental setup used in proof-of-concept demonstration of optical data compression via warped time stretch imaging. A train of broadband optical pulses were generated at 1550 nm central wavelength with a repetition rate of 36.129 MHz and a pulse width slightly less than 100 fs. The laser pulses were temporally stretched to about 1 ns by a dispersion compensating fiber and amplified by an erbium-doped fiber amplifier (EDFA). The bandwidth over 1541 nm to 1561 nm was selected by a wavelength division multiplexing (WDM) filter. The pulses passed through an optical circulator and were coupled to free-space part of STEAM setup with a fiber collimator. There, a pair of diffraction gratings generates a one-dimensional rainbow with each wavelength component imaging a different location at the target. The spectrally-encoded rainbows are reflected and coupled back into the fiber, carrying the image information. The nonuniform space-to-frequency-to-time mapping is achieved with a warped chirped fiber Bragg grating (CFBG). After optical image amplification by another EDFA, different wavelength components are detected serially by a single-pixel photodetector and acquired by an analog-to-digital converter (ADC).

The experimental setup used for our proof-of-principle demonstration of optical image compression is shown in Fig 4. A mode-locked fiber laser generated pulses at around 1550 nm with a repetition rate of 36.129 MHz and a pulse width slightly less than 100 fs. A short dispersion compensating fiber with an overall dispersion of 10 ps/nm was used to temporally broaden pulses to about 1 ns, so that an erbium-doped fiber amplifier (EDFA) can amplify them without any distortion. Since the output spectrum of EDFA is sensitive to the input polarization, a polarization controller was used to change the polarization of the input pulses to EDFA. The polarization was tuned in such a way that the output amplified pulses had relatively symmetric spectrum around 1550 nm. Amplified pulses then entered a coarse wavelength-division multiplexing (WDM) filter, and the output of 1551 nm channel was used to shape laser pulses with a considerably low noise floor over 1541 nm to 1561 nm bandwidth. These pulses passed through an optical circulator and were coupled to free-space with a fiber collimator.

**Fig 5:**
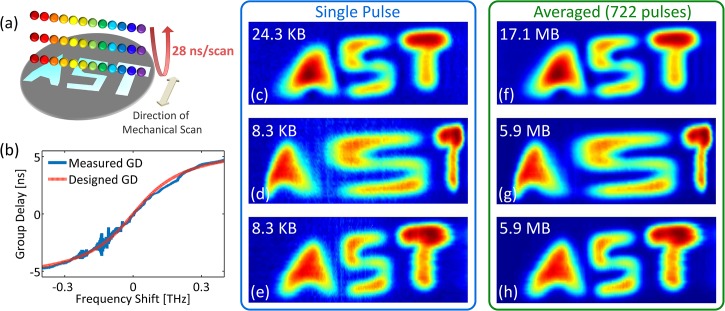
Proof-of-concept experimental results. (a) The test sample reflected one-dimensional rainbow illumination pulses, which are used to perform time stretch imaging at a scan rate of 36 MHz. The field of view, determined by the length of the rainbow was 5 cm and covers the width of the target. The vertical direction was scanned by mechanical translation at 0.5 mm per step. (b) The warped stretch transform leading to nonlinear spectrum-to-time mapping is performed by a custom chirped fiber Bragg grating with sublinear group delay (GD) profile. This profile gives higher group delay dispersion at the center frequency and reduced dispersion at the wings of the bandwidth. (c) If we use a linear group delay profile with the same dispersion as that of the warped stretch at the center frequency and a single pulse per image line, the image data size would be 24.3 KB (553×45 measured pixels). (d) The single-pulse reconstructed image based on the waveform nonlinearly stretched by the chirped fiber Bragg grating has an obvious warping effect at the center of the field of view (letter “S”) (189×45 measured pixels). (e) The single-pulse unwarped reconstructed image data size is 8.3 KB achieving about 3 times optical image compression (189×45 measured pixels). (f, g and h) When many pulses (722 pulses here) are averaged to form each horizontal line image, the images quality improve only slightly over Fig 5c, 5d and 5e, proving high signal-to-noise ratio of our camera even in single-pulse capture mode. The number of measured pixels used in formation of Fig 5f is 722×553×45, and for Fig 5g and 5h is 722×189×45. The temporal durations of the waveforms corresponding to each horizontal line in images 5c-5h are 27.7 ns, 9.5 ns, 9.5 ns, 20.0 μs, 6.8 μs, and 6.8 μs, respectively.

Free-space laser pulses were linearly polarized with quarter- and half-wave plates, and then, they were spatially dispersed with a pair of reflection diffraction gratings, so that each wavelength component of the collimated beam was positioned at a different lateral point similar to a rainbow. The width of the rainbow depended on the size of the second diffraction grating and the distance between two diffraction gratings, and the height of the rainbow depended on the beam size from the fiber collimator. In this setup, the total bandwidth of the pulses interrogating the target is limited to about 10 nm centered at 1551 nm because of the clipping of the rainbow at the edges of the second diffraction grating. The horizontal field of view, which was dictated by the width of the rainbow, was 5 cm. Different wavelength components of the rainbow reached a reflective object. Each pulse of the mode-locked laser generates one rainbow, which captures one line image across the field of view. The rainbow components located at the target were reflected back (Fig 5A) and returned all the way back to the fiber, where they were directed with the optical circulator to an amplified time-stretch system (Fig 4). The nonlinearly dispersed pulses with chirped group delay profile are captured by a 10 GHz-bandwidth single-pixel photodetector and digitized in real-time. An analog-to-digital convertor (ADC) with a sampling rate of 20 GSps and 7 GHz bandwidth was used to digitize the output signal of the photodetector. To achieve warped stretch, we used a fiber Bragg grating with customized chirp profile, whose performance was studied in [20] and is shown in Fig 5B.

The two-dimensional image was reconstructed by stacking spectrally-encoded horizontal line images at different steps of the vertical scan. If instead of the fiber Bragg grating, a dispersive fiber with linear group delay was used, the reconstructed image from one pulse per horizontal line was as shown in Fig 5C. But, for the case of a fiber Bragg grating, since each line-scan is warped, the warping of the image is observed in the horizontal direction (Fig 5D). This effectively means that the central area (letter “S”) is sampled with higher resolution than the peripherals (letters “A” and “T”). With the unwarping algorithm derived from the reverse dispersion profile, the uniform image was successfully reconstructed with a reduced data acquisition time and number of samples (Fig 5E). Compared to the case of the linear group delay (Fig 5C), an image with comparable quality is generated with only one-third of the data size (Fig 5E). We note that the reconstruction is an intensity-only operation and does not require optical phase retrieval. Images with improved quality can be generated by averaging many pulses to form each horizontal line image. However, this reduces the frame rate of the time stretch camera. Fig 5F, 5G, and 5H show such images formed by averaging 722 pulses for each horizontal line. Although the image quality is slightly better using averaging, but in our demonstration, the signal-to-noise ratio even in single-pulse acquisition mode (Fig 5C, 5D, and 5E) is high enough that the target features are clearly recognizable, and there is no need for averaging. This is due to the relatively high pulse-to-pulse stability of the STEAM setup.

**Fig 6:**
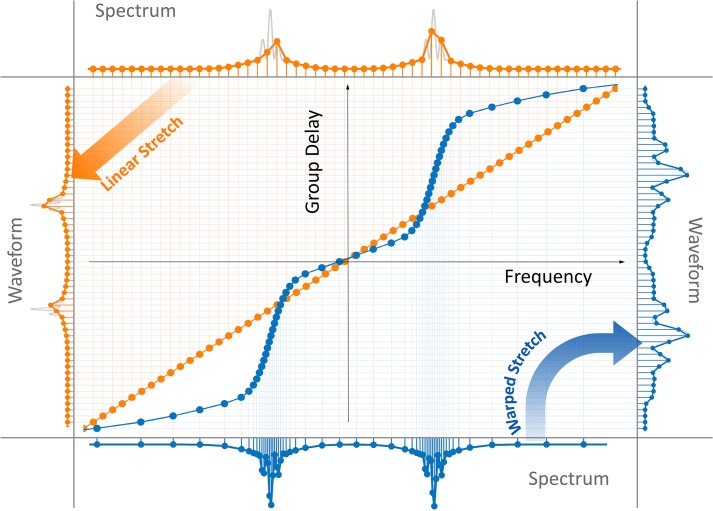
Complex nonlinear stretch transforms. A nonlinear group delay profile with higher dispersion (slope) at two regions of the spectrum (blue plots) than the linear profile (orange plots), leads to a higher spectral resolution in these two regions (compare the spectrums). However, the resolution is reduced at the wings and the center of the spectrum, where information is sparse. This improves the image quality at two corresponding regions of the field of view by sacrificing the quality at the sparse peripheral and central regions. Since the total group delay of linear and nonlinear profiles are the same, the file size determined by the acquisition time and the overall number of samples in the waveform remains unchanged. The gray curves show the analog waveforms before sampling for the purpose of comparison.

Warped group delay profiles used here are only a few cases of the unlimited variety of nonlinear space-to-frequency-to-time mappings that can be integrated into time stretch imaging, each corresponding to their unique nonuniform sampling patterns. As another example, Fig 6 shows the nonlinear frequency-to-time mapping profile that is designed for a microfluidic channel with two focal regions (cell flow lanes), a common case in inertial focusing [36, 37]. The profile shown here is designed to have two high-resolution sampling areas corresponding to where the cells are confined. Three low-resolution sampling regions provide coarse resolution in the peripherals regions between the cell flow lanes. The nonuniform mappings can even be reshaped dynamically based on relatively slower transitions in the sparsity characteristics of the image, in other words, alterations in the information rich areas of the image. To achieve such a functionality, the group delay profile of the dispersive element should be tunable and controlled by a feedback mechanism. In terms of tunable dispersion, Chromo-Modal Dispersion (CMD) offers wide tunability, broad spectrum, and low loss [38].

Furthermore, warped stretch imaging is not limited to using warped group delay to perform nonlinear space-to-frequency-to-time mapping. It can also be achieved by nonuniform space-to-frequency mapping, eg. warped rainbows, where rainbow frequency components are not equally spaced. This can be implemented by frequency-dependent spatial dispersers such as custom-designed diffraction gratings and virtually imaged phased arrays.

## Conclusion

Real-time optical image compression is needed to address the fundamental challenges in acquiring and storing the large amount of data generated in high-speed imaging. Here, we have demonstrated one such technique applied to time stretch imaging. Using warped group delay dispersion, we achieved warped stretch imaging in such a way that the information-rich central vision is sampled at a higher sample density than the sparse peripheral vision. Most notably, this was done using a uniform electronic sampler, i.e. without adaptive or dynamic control over the electronic sampling rate. A three-time image compression was achieved in experimental proof of concept demonstration. Our nonuniform sampling technique could offer one route to taming the capture, storage, and transmission bottlenecks associated with big data.

## References

[1] Jalali B, Asghari MH. Anamorphic Stretch Transform: Putting the squeeze on Big Data. Opt Photonics News. 2011; 5 (2): 24–31.

[2] Goda K, Tsia KK, Jalali B. Serial time-encoded amplified imaging for realtime observation of fast dynamic phenomena. Nature. 2009; 458: 1145–1149. 10.1038/nature07980 19407796

[3] Mahjoubfar A, Chen CL, Niazi KR, Rabizadeh S, Jalali B. Label-free high-throughput cell screening in flow. Biomed Opt Express. 2013; 4: 1618–1625. 10.1364/BOE.4.001618 24049682 PMC3771832

[4] Mahjoubfar A, Goda K, Ayazi A, Fard A, Kim SH, Jalali B. High-speed nanometer-resolved imaging vibrometer and velocimeter. Appl Phys Lett. 2011; 98: 101107.

[5] Mahjoubfar A, Goda K, Wang C, Fard A, Adam J, Gossett DR, et al 3D ultrafast laser scanner. Proc SPIE. 2013; 8611: 86110N.

[6] Mahjoubfar A, Chen CL, Niazi K, Rabizadeh S, Jalali B. Label-free high-throughput imaging flow cytometry. Proc SPIE. 2014; 8972: 89720F.

[7] Chen CL, Mahjoubfar A, Huang A, Niazi K, Rabizadeh S, Jalali B. Hyper-dimensional analysis for label-free high-throughput imaging flow cytometry. Conf Lasers Electro Optics. 2014; Applications and Technology: AW3L.2.

[8] Qian F, Song Q, Tien EK, Kalyoncu SK, Boyraz O. Real-time optical imaging and tracking of micron-sized particles. Opt Commun. 2009; 282 (24): 4672–4675.

[9] Wong TTW, Lau AKS, Ho KKY, Tang MYH, Robles JDF, Wei X, et al Asymmetric-detection time-stretch optical microscopy (ATOM) for ultrafast high-contrast cellular imaging in flow. Sci Rep. 2013; 4: 3656. 10.1038/srep03656 PMC3888978 24413677

[10] Lau AKS, Wong TW, Ho KY, Tang MTH, Chan ACS, Wei X, et al Interferometric time-stretch microscopy for ultrafast quantitative cellular and tissue imaging at 1 μm. J Biomed Opt. 2014; 19 (7): 076001. 10.1117/1.JBO.19.7.076001 24983913

[11] Zhang C, Xu Y, Wei X, Tsia KK, Wong KKY. Time-stretch microscopy based on time-wavelength sequence reconstruction from wideband incoherent source. Appl Phys Lett. 2014; 105: 041113.

[12] Wei X, Lau AKS, Xu Y, Zhang C, Mussot A, Kudlinski A, et al Broadband fiber-optical parametric amplification for ultrafast time-stretch imaging at 1.0μm. Opt Lett. 2014; 39: 5989–5992. 10.1364/OL.39.005989 25361137

[13] Diebold ED, Buckley BW, Gossett DR, Jalali B. Digitally synthesized beat frequency multiplexing for sub-millisecond fluorescence microscopy. Nat Photonics. 2013; 7: 806–810.

[14] Solli DR, Ropers C, Koonath P, Jalali B. Optical rogue waves. Nature. 2007; 450: 1054–1057. 18075587 10.1038/nature06402

[15] Goda K, Ayazi A, Gossett DR, Sadasivam J, Lonappan CK, Sollier E, et al High-throughput single-microparticle imaging flow analyzer. Proc Natl Acad Sci U S A. 2012; 109 (29): 11 630–11 635. 10.1073/pnas.1204718109 PMC3406874 22753513

[16] Ng W, Rockwood T, Reamon A. Demonstration of Channel-Stitched Photonic Time-Stretch Analog-to-Digital Converter with ENOB ≥ 8 for a 10 GHz Signal Bandwidth. GOMACTech-14, Charleston, South Carolina, 3/31/2014-4/3/2014.

[17] Goda K, Mahjoubfar A, Wang C, Fard A, Adam J, Gossett DR, et al Hybrid Dispersion Laser Scanner. Sci Rep. 2012; 2:445 10.1038/srep00445 22685627 PMC3370333

[18] Yazaki A, Kim CK, Chan J, Mahjoubfar A, Goda K, Watanabe M, et al Ultrafast dark-field surface inspection with hybrid-dispersion laser scanning. Appl Phys Lett. 2014; 104 (25): 251106.

[19] Asghari MH, Jalali B. Anamorphic transformation and its application to time bandwidth compression. Appl Opt. 2013; 52: 6735–6743. 10.1364/AO.52.006735 24085172

[20] Asghari MH and Jalali B. Experimental demonstration of real-time optical data compression. Appl Phys Lett. 2014; 104: 1–4.

[21] Jalali B, Chan J, Asghari MH. Time bandwidth engineering. Optica. 2014; 1: 23–31.

[22] Chan J, Mahjoubfar A, Asghari MH, Jalali B. Reconstruction in Time-Bandwidth Compression Systems. Appl Phys Lett. 2014; 105 (22), 221105.

[23] Goda K, Jalali B. Dispersive Fourier transformation for fast continuous single-shot measurement. Nat Photonics. 2013; 7: 102–112.

[24] Mahjoubfar A, Goda K, Betts G, Jalali B. Optically amplified detection for biomedical sensing and imaging. J Opt Soc Am. 2014; 30 (10): 2124–2132. 10.1364/JOSAA.30.002124 24322867

[25] Chen H, Weng Z, Liang Y, Lei C, Xing F, Chen M, et al. High speed single-pixel imaging via time domain compressive sampling. Conf Lasers Electro Optics. 2014; OSA Technical Digest: JTh2A.132.

[26] Bosworth BT, Foster MA. High-speed flow imaging utilizing spectral-encoding of ultrafast pulses and compressed sensing. Conf Lasers Electro Optics. 2014; OSA Technical Digest: ATh4P.3.

[27] Nichols JM, Bucholtz F. Beating Nyquist with light: a compressively sampled photonic link. Opt Express. 2011; 19: 7339–7348. 10.1364/OE.19.007339 21503044

[28] Valley GC, Sefler GA, Shaw TJ. Compressive sensing of sparse radio frequency signals using optical mixing. Opt Lett. 2012; 37: 4675–4677. 23164876 10.1364/ol.37.004675

[29] Bosworth BT, Foster MA. High-speed ultrawideband photonically enabled compressed sensing of sparse radio frequency signals. Opt Lett. 2013; 38: 4892–4895. 10.1364/OL.38.004892 24322159

[30] Liang Y, Chen M, Chen H, Lei C, Li P, and Xie S. Photonic-assisted multi-channel compressive sampling based on effective time delay pattern. Opt Express. 2013; 21: 25700–25707. 10.1364/OE.21.025700 24216795

[31] Chen Y, Yu X, Chi H, Jin X, Zhang X, Zheng S, et al Compressive sensing in a photonic link with optical integration. Opt Lett. 2014; 39: 2222–2224. 10.1364/OL.39.002222 24978956

[32] Gupta V, Jafferji I, Garza M, Melnikova VO, Hasegawa DK, Pethig R, et al ApoStream, a new dielectrophoretic device for antibody independent isolation and recovery of viable cancer cells from blood. Biomicrofluidics. 2012; 6(2): 024133. 10.1063/1.4731647 PMC3396706 23805171

[33] http://commons.wikimedia.org/wiki/File:Istv%C3%A1n_Orosz_cylinder_painting.jpg.

[34] Shapiro HM. Practical flow cytometry 4th ed. John Wiley & Sons; 2003.

[35] Adam J, Mahjoubfar A, Diebold ED, Buckley BW, and Jalali B. Spectrally encoded angular light scattering. Opt Express. 2013; 21 (23): 28960–28967. 10.1364/OE.21.028960 24514410

[36] Di Carlo D, Irimia D, Tompkins RG, Toner M. Continuous inertial focusing, ordering, and separation of particles in microchannels. Proc Natl Acad Sci U S A. 2007; 104: 18892–7 18025477 10.1073/pnas.0704958104 PMC2141878

[37] Di Carlo D. Inertial microfluidics. Lab Chip. 2009; 9: 3038–3046 10.1039/b912547g 19823716

[38] Diebold ED, Hon NK, Tan Z, Chou J, Sienicki T, Wang C, et al Giant tunable optical dispersion using chromo-modal excitation of a multimode waveguide. Opt Express. 2011; 19: 23809–23817. 10.1364/OE.19.023809 22109406

